# Efficacy of electrical acupuncture on vascular cognitive impairment with no dementia: study protocol for a randomized controlled trial

**DOI:** 10.1186/s13063-018-2458-1

**Published:** 2018-01-19

**Authors:** Tian Li, Huangan Wu, Franscisca Soto-Aguliar, Li Huang, Wentao Li, Lixing Lao, Shifen Xu

**Affiliations:** 1 Shanghai Municipal Hospital of Traditional Chinese Medicine, Shanghai University of Traditional Chinese Medicine, Shanghai, 200071 China; 2 Shanghai Research Institute of Acupuncture and Meridian, Shanghai University of Traditional Chinese Medicine, Shanghai, 200030 China; 3 University of Maryland School of Medicine, Baltimore, MD 21201 USA; 4 School of Chinese Medicine, The University of Hong Kong, Hong Kong, China

**Keywords:** Vascular cognitive impairment with no dementia (VCIND), Vascular dementia (VD), Electrical acupuncture

## Abstract

**Background:**

Vascular cognitive impairment with no dementia (VCIND), manifested mainly as mild impairment of concentration and executive function, is the early phase of vascular dementia (VD). Currently, there is no specific treatment for VCIND. We hypothesize that electrical acupuncture can improve the mental and motor functions of patients with VCIND. Thus, we designed this randomized controlled trial to test this hypothesis by comparing the therapeutic effect of electrical acupuncture versus sham acupuncture in patients with VCIND.

**Method/Design:**

In this single-center 3-year study, 120 eligible patients will be recruited and randomly assigned to receive electrical acupuncture treatment (n = 60) or sham acupuncture (n = 60) for 8 consecutive weeks (24 sessions in total), with the same acupoint prescription (DU20, EX-HN3, DU24, DU17, DU26, EX-HN1, HT7, PC6, GB20, SP6). The primary assessment is the Montreal Cognitive Assessment. The secondary assessments are the Modified Barthel Index and Event-Related Potential. All outcomes will be assessed at baseline, endpoint, and follow-up at 8 and 24 weeks after the end of treatment.

**Discussion:**

If the outcome confirms the effectiveness and safety of electrical acupuncture in treating VCIND, this treatment is expected to be promoted in clinical practice to treat such patients.

**Trial Registration:**

Chinese Clinical Trial Registry identifier: ChiCTR-IIR-17011513; Registered on 27 May 2017.

**Electronic supplementary material:**

The online version of this article (10.1186/s13063-018-2458-1) contains supplementary material, which is available to authorized users.

## Background

Although global stroke incidence and mortality declined from 1999 to 2013, the absolute number of people affected by stroke continues to increase rapidly [1], with up to 41% of them suffering from varying degrees of cognitive impairment in the first year after the stroke [2], with ischemic stroke likely facilitating the onset of dementia [3]. Deb et al. [4] studied the costs of caring for people with dementia, finding that it has been a great social burden in the United States from 2006 until 2017. Among all types of dementia, vascular dementia (VD) is the second most common type after Alzheimer’s disease (AD), accounting for 10% to 50% of the entire demented population [5].

Vascular cognitive impairment with no dementia (VCIND) is the early phase of VD, and manifests mainly as impairment of concentration and executive function [6, 7]. The presence of VCIND means that the patient’s current cognitive function has been impaired and implies further deterioration. However, with timely diagnosis and following effective treatment, the progression of VCIND, and even its development into VD, can be delayed. Furthermore, cognitive function is one of the essential factors that influence the prognosis of stroke rehabilitation. Thus, together with the activities of daily living, improving cognitive function is very important in the overall rehabilitation process for stroke patients [8].

Electrical acupuncture therapy coordinates the balance of yin and yang, and regulates the organs’ functions and mental status [9]. In fact, previous research on electrical acupuncture treating mental diseases have revealed its advantages, including relieving symptoms, reducing adverse reactions to long-term medication, and improving their curative effect, as well as delaying the progression of disease [10, 11]. There are studies that support the therapeutic effect of electrical acupuncture therapy on alleviating the severity of cognitive impairment and improving the quality of life of patients [12, 13]; however, most of these studies are empirical [14]. The deficit of objectivity makes the findings of those reports far from persuasive and thus limits the common application of acupuncture for VCIND. Therefore, through this proposed randomized controlled trial (RCT), we intend to obtain better evidence for the use of electrical acupuncture in the treatment of VCIND.

## Methods and design

### Hypothesis

Participants with VCIND who receive electrical acupuncture treatment will have an improvement in their mental and motor functions.

### Objectives

1. To compare the difference in improvement of cognitive function and mental states, assessed by the Montreal Cognitive Assessment (MoCA), between the intervention group and control group.
2. To compare the difference in improvement of daily-life ability, measured by the Modified Barthel Index (MBI), between the intervention group and control group.
3. To compare the difference in improvement of Event-Related Potential (ERP) abnormality between the intervention group and control group.

### Design

**Fig. 1:**
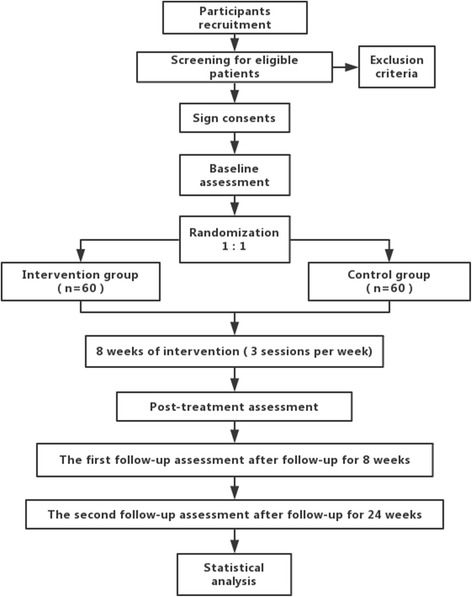
Study process. Shows the flowchart of patients through the study process

**Fig. 2:**
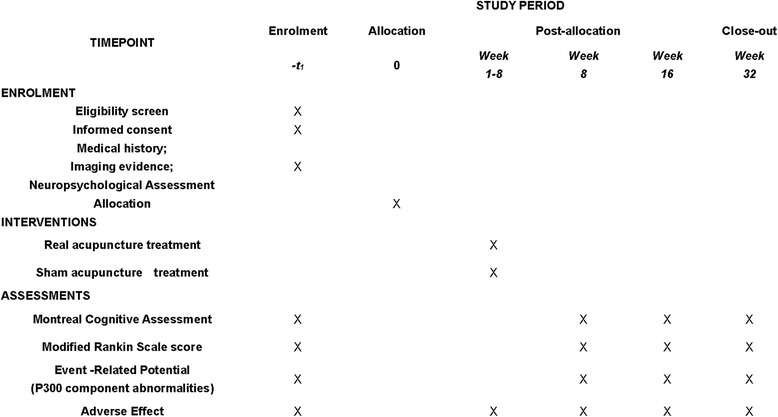
Standard Protocol Items: Recommendations for Interventional Trials Statement (SPIRIT) Figure. Shows the enrolment, interventions, and data collection

We developed this single-center, single blind RCT according to the Standard Protocol Items: Recommendations for Interventional Trials Statement. The participant flowchart can be found in Fig. 1 and details of study process and data collection will be elaborated in Fig. 2 and Additional file 1.

### Methods

Ethical approval has been obtained from the ethics committee of Shanghai Municipal Hospital of Traditional Chinese Medicine affiliated with Shanghai Traditional Chinese Medicine University in March 2017.

A recruitment notice directed to post-stroke patients with cognitive impairment and their companions will be posted on an official information platform or bulletin boards at Shanghai Municipal Hospital of Traditional Chinese Medicine in Shanghai, China. Any interested patients, both as in-patients or out-patients, can reach the researchers through the phone number provided. After being informed of the details of this RCT, voluntary participants will be evaluated by experts in the neurological department of this hospital, and those that meet the selection criteria will be asked to sign the informed consent (Additional file 2) before being accepted into this RCT.

#### Inclusion criteria

Patients aged 45–80 years who (1) complain of or are reported by a relative or accompanying person as having cognitive decline; (2) have imaging evidence that supports cerebrovascular disease, and may or may not have a medical history of transient ischemic attack or other types of stroke; (3) have symptoms or signs caused by cerebrovascular disease (e.g., hemiplegia, hemiparesis, problems with understanding or forming speech, numbness or strange sensations); and (4) obtain the following results in the neuropsychological assessment under professional instruction from the neurologist: MoCA score < 26; clinical dementia rating scale score = 0.5; modified Rankin scale score ≤ 3; Hachinski ischemic score > 7 points, will be included in the study.

#### Exclusion criteria

Patients who (1) have a Mini-mental State Examination score < 21; (2) have co-morbidity including AD, Parkinson’s disease, frontotemporal dementia, or Huntington’s disease, central nervous system inflammatory demyelinating disease, epilepsy, psychosis, liver or kidney dysfunction, hypothyroidism, alcoholic or drug abuse related encephalopathy, depression; and (3) have severe visual impairment, hearing impairment, severe aphasia or limb dysfunction which might affect pre-assessment, will be excluded from the study.

#### Dropout criteria

Patients who (1) have poor clinical compliance (2) quit the RCT voluntarily will be considered as having dropped out.

In this procedure, we used the formula for calculating the sample size of two sample rates for disordered classified data. Taking the results of previous research with a similar study design of acupuncture arm [15] (RR = 80%, OR = 15%) into consideration, a sample size of 100 patients (50 for each group) should be recruited. Allowing 20% attrition, the recruited sample size for this RCT will be of 120 patients (60 in each group).

Upon fulfillment of selection criteria, 120 eligible participants will be randomly allocated into the intervention and control groups in a 1:1 allocation ratio. Randomization will be performed according to a random list of numbers generated by the randomization center of Shanghai University of Traditional Chinese Medicine. An independent researcher, who contacts no participant and is not involved in the data collection or analysis, will take charge of the participant allocation based on the mentioned random list as well as on the allocation sequence concealment.

All participants will receive moderate financial subsidies to cover the travel expenses and should stick to the schedule for all treatments and assessments as much as possible. Those who are unable to visit the clinic at the time of reservation should inform the instructor by phone in advance to reschedule.

Patients will receive their intervention in a personal space separated by screens and no communication between them will be allowed. Treatment will be conducted in 8 consecutive weeks, at a frequency of three sessions per week in both intervention and control groups, and all manipulation should be consistent with the standards of the Revised Standards for Reporting Interventions in Clinical Trials of Acupuncture (STRICTA) [16]. In both groups, a 30 × 60-cm plastic plate with a notch will be applied on the patient’s chest as a visual barrier. In each session, after skin sterilization with alcohol wipes, the licensed and experienced acupuncturists will apply, with or without needle insertion, 0.25 × 25-mm needles to acupoints DU20, EX-HN3, DU24, DU26, and EX-HN1 and 0.30 × 40-mm needles for acupoints DU17, HT7, PC6, GB20, and SP6. A 2 × 2-cm piece of tape will be used to affix each needle to the skin of the chosen acupoints. The needles at DU20 and EX-HN3 will be connected to a G6805-2 Multi-Purpose Health Device (Shanghai Medical Instruments High-Techno, China) with electrode clamps. It will be implied to all patients that the sensation they are experiencing is the real acupuncture sensation. After a 30-min retention period, all needles and tape will be removed.

The intervention group will have a real tube-needle insertion applied with disposable stainless steel needles (Wuxi Jiajian Medical Material Co., Ltd., Wuxi, China). In the sitting position, DU17 will be punctured obliquely towards jaw to a depth of 0.5 cun into the skin. After that, in the supine position, DU20 (with the needle tip pointing towards the ground), EX-HN3, DU24, DU26, EX-HN1, and GB20 (with the needle tip pointing towards the feet) will be punctured obliquely to a depth of 0.5 cun, while HT7 and SP6 (with the needle tip pointing towards the limb extremities) will be punctured perpendicularly to the respective depth of 0.5 cun and 1–1.5 cun. Appropriate needle manipulation (including lifting, thrusting, and rotating) will be applied for 10 seconds on each point so that the needling sensation (De-qi sensation) can be accomplished at each point.

The control group will undergo treatment with a special sham acupuncture device with a blunt-tip needle. This type of needle can provoke a needling sensation quite similar to real acupuncture as soon as it touches the skin, without insertion. The acupuncturists should pretend to do the needle manipulation for 10 seconds on each point as well. Similarly, the needles on DU20 and EX-HN3 will be connected to a G6805-2 Multi-Purpose Health Device, with the device switched off, and will remain in place for the next 30 minutes.

The details of interventions are elaborated on Table 1.

**Table 1:** Details of intervention

	Intervention group	Control group
Acupoints	DU20, EX-HN3, DU24, DU26, EX-HN1, DU17, HT7, PC6, GB20, SP6	DU20, EX-HN3, DU24, DU26, EX-HN1, DU17, HT7, PC6, GB20, SP6
Depth of insertion	DU20, DU17, EX-HN3, DU24, DU26, EX-HN1, GB20, HT7; 10 mm SP6; 15 mm	No insertion
Needle type	Steel needles (Wuxi Jiajian Medical Material Co., Ltd., Wuxi, China)	Blunt-tip needle (Streitberger Placebo-needle)
Needle sensation	With de-qi sensation	Without de-qi sensation
Electric stimulation	Needles on DU20 and EX-HN3 connected to G6805-2 Multi-Purpose Health Device (Shanghai Medical Instruments High-Techno, China), with electric pulse at a frequency of 2.5 Hz and an intensity of 45 mA	Needles on DU20 and EX-HN3 connected to G6805-2 Multi-Purpose Health Device (Shanghai Medical Instruments High-Techno, China), without electric pulse
Frequency and duration	Three sessions per week for 8 weeks	Three sessions per week for 8 weeks

### Outcome measures

All assessments will be conducted at baseline, endpoint (8 weeks after treatment commencement), and follow-up (8 weeks and 24 weeks after the end of treatment). All questionnaires are in Chinese [17].

#### Primary outcome measure

The primary outcome measure includes changes of MoCA score. The MoCA test is a special tool to assess eight cognitive domains, including short-term memory recall, visuospatial abilities, language, attention, working memory, orientation to time and place, concentration as well as executive functions, among which the last two are the most notable aspects for VCIND. Executive functions are assessed by the alternating trail making task, verbal fluency task and the two-item verbal abstraction task; while concentration is evaluated by a sustained attention task, a serial subtraction task and digits forward and backward task. A final score ≥ 26 usually indicates normal cognitive ability, while a score below that level indicates cognitive impairment. This test will be administrated in approximately 10 minutes.

#### Secondary outcome measures

The ability of daily life will be evaluated by using the MBI. It involves 10 variables and a higher score indicates a greater likelihood of being able to live at home independently [18].

ERP provides a non-invasive way to evaluate brain function in patients with cognitive impairment by observing the typical electrophysiological response to a stimulus. In this RCT, it is the P300 component abnormalities (change of latent period and wave amplitude) that will be observed [19].

### Blinding

Except for the acupuncturists, patients and researchers who measure the outcomes will be blinded to the randomization in this RCT.

### Safety assessment

All participants are asked to report any adverse event related to the treatment they are undergoing to the specific researcher in charge of assessing adverse effects. The details of every adverse event, including following management and final outcome, will be reported in the Case Report Form.

### Monitoring

The entire process of this RCT will be conducted under the supervision of a qualified clinical trial expert, and all original data of the RCT will be inputted into and stored on the ResMan research Manager of the Clinical Trial Management Public Platform. The Clinical Research Center of Drugs of the Shanghai University of Traditional Chinese Medicine takes charge of data monitoring, i.e., having access to any interim results and making the final decision to terminate the trial.

### Data analysis

All original data will be entered and stored with Microsoft Excel 2010, while data from cases without baseline evaluation or follow-up will be excluded from the full analysis set, and analyses will be performed with SPSS version 23.0 software. In the statistical analysis, measurement data will be analyzed with the Student’s *t* test, ranked data with the rank sum test, and categorical data with χ^2^ test. Data will be expressed as mean ± standard deviation or median (first quartile, third quartile). All reported *P* values will be two-sided with confidence intervals at the 95% level, and a *P* value of less than 0.05 will be considered statistically significant.

## Discussion

In the past few years, acupuncture therapy has been used in clinical practice on VCIND for relieving symptoms and delaying progression. However, the existing literature regarding those practices are basically empirical and with inevitable deficits such as small sample size or short duration [20]. In this trial, we used the study by Lao et al. [21], which reported the successful implementation of sham acupuncture, as a reference and will perform the control-group intervention, namely sham acupuncture, to rule out the placebo effect of acupuncture in a very rigorous way. By designing this RCT – with an improved design and methods – we aim to contribute better evidence for the effectiveness of electrical acupuncture, which is expected to reduce the extent of damage to nerve cells in the hippocampus, leading to a neuro-protective effect [22] in vascular cognitive impairment.

The second improvement we make in this study is the selection of participants. Symptoms like mild functional impairment for complex tasks can typically be apparent in mild cognitive impairment, early single dementia, or even multiple etiology dementias [23]. Thus, it is clinically difficult to identify patients with pure VCIND, and results based on that population might not be generalizable to clinical practice. To avoid this problem, we chose to target early cognitive impairment populations or pre-VCIND populations, rather than those clearly diagnosed as vascular cognitive impairment.

Finally, to promote good patient compliance, we will try to facilitate each step of the trial, including the appointment process, treatment conduction, and phone call or home visiting follow-up, as well as offering moderate financial subsidies.

### Trial status

We will start recruiting participants in November 2017.

## Additional files

Additional file 1: The SPIRIT checklist. (DOC 107 kb)

Additional file 2: The informed consent form. (DOCX 15 kb)

## Abbreviations

AD: Alzheimer’s disease
ERP: event-related potential
MBI: Modified Barthel Index
MoCA: Montreal Cognitive Assessment
RCT: randomized controlled trial
STRICTA: Standards for Reporting Interventions in Clinical Trials of Acupuncture
VCIND: vascular cognitive impairment with no dementia
VD: vascular dementia
