# Supplementary material for: Efficacy of electrical acupuncture on vascular cognitive impairment with no dementia: study protocol for a randomized controlled trial
Source: Trials. 2018 Jan 19;19:52. doi: 10.1186/s13063-018-2458-1 (PMC5775601; doi:10.1186/s13063-018-2458-1)
Supplement: Supplementary file 2 — The informed consent form. (DOCX 15 kb) [file 13063_2018_2458_MOESM2_ESM.docx]

**Informed Consent Form**

Name of participant: Gender: Age: Registry No:

Dear participant:

This study is set to investigate the therapeutic effect of electrical acupuncture in patients with ***vascular cognitive impairment with no dementia (VCIND)***.

This is a clinical randomized controlled trial. If you agree to participate, you will be randomly assigned to an acupuncture intervention group or a sham acupuncture group. Therapies in both groups are relatively safe. If any unfavorable or unintended signs, symptoms, or diseases occur, you should report them to the doctors and they will take a positive response.

During the course of the study, you will need to: (1) complete the entire course of treatment (24 treatments) by the acupuncturist, (2) during the study, you will fill out some questionnaires and answer some questions to evaluate the efficacy of the treatment regimen (4 times, before treatment, endpoint of the treatment, 8 weeks and 24 weeks after the ending).

The participation in this study is entirely voluntary. Participants may be withdrawn at any time during the course of treatment without affecting the relationship between you and doctors. There is no loss in medical and economic aspects for you. You can also receive the effective treatment from doctors following routine medical procedures.

The research will strictly protect your privacy according to the principles of the Declaration of Helsinki. All information in this study will be kept confidential, and your private information will not appear in the research summary and published literature. This study has been ethically reviewed by the Ethics Committee of Shanghai Municipal Hospital of Traditional Chinese Medicine (2017SHL-KY-03).

**Voluntary Subject Statement**:

I have learned about the requirements of the clinical study in detail and the potential adverse reactions associated with it. I voluntarily participate in this clinical study, receive treatment on time, and fill out the relevant health questionnaire in accordance with the requirements of the research program. If adverse reactions occur, I will report to the doctor promptly. At the same time I know that the doctor will give positive response for my possible adverse reactions. I am also entitled to withdraw from the study at any time for any reason. However, if there are no special circumstances, I will cooperate with the doctor to complete the clinical study. My participation and the personal data in the trial are confidential. I agree with my doctor, the relevant regulatory authorities, and the ethics committee to review my information as required.

I (signature) relative (signature) (Relationship) Date: D M Y

**Doctor's declaration**

I have fully explained the requirements of the clinical study in detail and the potential adverse reactions to the above participant/relative, and answered their questions. To the best of my knowledge, the participant/relative has been informed adequately and has consented.

Doctor’s signature: Date: D M Y

*In the event of inconsistency or discrepancy between the Chinese version and the English version, the Chinese language version shall prevail.*
